# Effectiveness of Therapeutic Gardens for People with Dementia: A Systematic Review

**DOI:** 10.3390/ijerph18189595

**Published:** 2021-09-12

**Authors:** Veronica Murroni, Raffaele Cavalli, Andrea Basso, Erika Borella, Chiara Meneghetti, Andrea Melendugno, Francesca Pazzaglia

**Affiliations:** 1 Department of General Psychology, Università degli Studi di Padova, 35121 Padova, Italy; veronica.murroni@studenti.unipd.it (V.M.); erika.borella@unipd.it (E.B.); chiara.meneghetti@unipd.it (C.M.); 2 Department of Land Environment Agriculture and Forestry, Università degli Studi di Padova, 35020 Padova, Italy; raffaele.cavalli@unipd.it; 3 “Giotto” Social Cooperative, 35127 Padova, Italy; andrea.basso@coopgiotto.com; 4 Casa Madre Teresa di Calcutta (O.P.S.A.), 35030 Padova, Italy; a.melendugno@operadellaprovvidenza.it; 5 Inter-University Research Center in Environmental Psychology (CIRPA), 00185 Roma, Italy

**Keywords:** therapeutic garden, horticultural therapy, Alzheimer’s disease, dementia, restorative environments, systematic review

## Abstract

This paper is a systematic review of quantitative studies conducted on the benefits of visiting gardens and gardening therapy for people with dementia (PWD) in an effort to assess the effectiveness of such treatments and obtain information on the most appropriate garden design for this population. This review followed the Preferred Reporting Items for Systematic Review and Meta-Analysis Protocols (PRISMA) guidelines. Four databases were searched (PubMed, Web of Science, PsycINFO, Scopus), with no time limits. Out of a total of 480 articles considered, 16 studies were selected for review. In all but two of the studies examined, gardening therapy and the use of therapeutic gardens induced psychophysiological improvements in PWD. The areas showing the greatest effects were Engagement, Agitation, Depression/Mood, Stress, and Medication. It also emerged that interest in this sphere has been growing in the last decade, but there is still a shortage of empirical evidence of the beneficial effects of therapeutic gardens in relation to the type and severity of dementia, and of garden design guidelines. Despite the limited number of studies investigated, the review confirmed the benefits of gardening and therapeutic gardens in PWD. There is nonetheless a need to conduct more quantitative research to support currently-available evidence and generate more information, focusing on garden design criteria, in-garden activities, the type and severity of dementia examined, and effects on caregivers as well as on PWD.

## 1. Introduction

### 1.1. Benefits of Contact with Nature

The positive effects of nature on human beings have their theoretical grounds in several bio-psycho-evolutionary approaches and theories that, starting in the 1980s, have promoted numerous empirical studies supporting the importance of contact with nature in improving people’s psychophysical wellbeing and quality of life. A first approach, called the biophilia construct, was proposed by Wilson [1]. It is based on the assumption that our species is instinctively attracted to the natural world. The word *biophilia*, deriving from Greek, literally means love of life. Two psychological theories justify the positive effects of contact with nature, one focusing on its physiological and affective effects, the other on its cognitive impact. Ulrich [2] developed a Stress Reduction Theory (SRT), according to which exposure to the natural world after an exhausting or threatening experience would promote psychophysiological recovery from its stressful effects. This regeneration mechanism would begin with a very rapid, positive affective response to certain environmental stimuli immediately identified by a human observer (an abundance of vegetation, the presence of water, animals, and so on). In an organism under stress, this first affective reaction triggers a physiological rebalancing mechanism: the parasympathetic system is activated, inducing a drop in physiological stress indices (cortisol level, heartbeat, and blood pressure). The psychological perception of stress decreases at the same time, as the related negative emotions fade and positive emotions replace them. A recent meta-analysis [3] conducted on 32 studies with 2356 participants confirmed the importance of contact with nature in promoting positive affective states and reducing stress. Another influential approach is called the Attention Restoration Theory (ART) [4]. Focusing on attention functions, it distinguishes between two attentional mechanisms [5]: voluntary attention, activated by the execution of complex cognitive tasks, which is liable to decay and needs to be restored; and involuntary attention, spontaneously captured by environmental properties, which is not subject to fatigue. ART suggests that the particular features of certain environments activate spontaneous attention, enabling voluntary attention to be restored at the same time. According to ART, there are four key components that characterize a restorative environment: fascination (the property of the environment to hold our attention with no voluntary effort); extent (the opportunity to feel immersed in the environment); being away (establishing a distance between us and our everyday routine); and compatibility (with our own inclinations). Fascination is considered the most important to the restoration process. As also confirmed by recent reviews [6], the natural environment (among others) has precisely these characteristics and can promote this process.

### 1.2. The Therapeutic Effects of Gardens and Horticulture

Contact with nature can be limited to exposure to greenery in general, or it can involve specific activities such as gardening therapy or the use of therapeutic gardens, both of which are among the non-pharmacological treatments recommended for PWD and other kinds of disease [7,8]. Therapeutic gardens can be used more or less actively, for gardening or other activities (e.g., psychotherapy), or passively (for walking or simply sitting in). They can be built inside or outside care facilities. They are defined as “therapeutic” because they are designed in such a way as to emphasize their curative potential. Sometimes the literature refers to “healing gardens” [9], where visitors can experience a lessening of their stress, and feel physically and mentally restored. Thaneshwari et al. [7] recommends that therapeutic gardens be designed specifically for the care of certain types of patient, such as Alzheimer’s gardens, or gardens for people with cancer. Söderback et al. [10] defined horticultural therapy as a gardening activity that includes interventions mediated by natural spaces such as gardens, using suitable tools, and proposing activities designed to suit a given type of patient. To be “therapeutic”, the purpose of the horticultural activity must be to promote the participants’ health and wellbeing. From a recent scoping review [11] of different therapies revolving around the use of natural elements (therapy with animals, horticulture, farming activities, presentations of natural stimuli in virtual reality), it emerged that 41 of the 85 studies identified had to do with gardening. They concerned different settings, including hospitals, retirement homes, and prisons, and reported positive results on several indexes of participants’ wellbeing. Studies on gardening and/or therapeutic gardens appear in several types of review dealing with different therapeutic activities and types of patient. Some reviews focused on a wide array of interventions involving the natural world [11,12,13]. Others examined general environmental interventions [14], sensory interventions [15], and interventions mediated by therapeutic gardens for various types of patient [7,16,17]. Some reviews focused specifically on horticulture [18,19,20], others more generally concerned outdoor spaces [21]. Some discussed a whole range of non-pharmacological interventions, including therapeutic gardens and gardening therapy [22]. Some were narrative reviews [23], or discussed the effects of therapeutic gardens and gardening therapy by drawing on qualitative studies [24]. A systematic review and meta-analysis [25] included five studies relating to horticulture in combination with other activities, and two studies that involved watching videos of natural scenery. Finally, a scoping review by Gonzalez and Kirkevold [26] examined the effects of the purposeful use of outdoor sensory gardens, gardening activities, and indoor plants in dementia care.

### 1.3. The Effects of Therapeutic Gardens and Horticultural Therapy on People with Dementia

The effects of therapeutic gardens on PWD are still being investigated. Recent reviews and studies [23,26,27] found that including therapeutic gardens in care environments has positive effects on agitation, behavior, walking, stress levels, self-esteem, depression, and aggressiveness. A quantitative review by Zhao et al. [20] on the benefits of gardening on PWD reported improvements in cognitive function, agitation, emotional state, and engagement, while no such effects on agitation or emotional state were obtained from visits to gardens or viewing nature scenes. For specific positive effects of gardening on patients with Alzheimer’s disease, see D’Andrea et al. [28], who found evidence of it promoting creativity, self-esteem, social interaction, sensory stimulation, gross and fine motor skills, and hand-eye coordination. Compared with the above-mentioned reviews, the present work focuses more specifically on dementia, therapeutic gardens, and gardening therapy, and only quantitative studies were considered. We explored the effects of therapeutic gardens and horticultural therapy on PWD in relation to the characteristics of the samples studied (severity of dementia), and the variables considered (behavior, affect, cognition). Our aims were to conduct a systematic review and critical analysis of the empirical support for these therapeutic approaches, and to suggest future directions to advance the field of such interventions for PWD. An added value compared with existing reviews lies in that the latest studies were included.

### 1.4. Objectives and Research Questions

This systematic literature review provides an update on research into the psychophysiological effects of gardening and therapeutic gardens on PWD. It sought to identify any differences in the therapeutic effect of gardens in terms of the different uses made of them, their physical features, and the severity of users’ dementia.

Our analysis of the published empirical evidence started with the following research questions:

1. How effective are therapeutic gardens for PWD?
2. Which domains (behavioral, cognitive, mood, sleep, physiological, etc.) are the most affected?
3. Which garden design features have the greatest effects (presence of water, types of plant, presence of animals, etc.)?
4. Which activities undertaken in the garden are the most effective (structured activities such as gardening or recreational activities such as doing physical exercises, spending time in the garden, or walking)?

## 2. Materials and Methods

### 2.1. Search Methods for Identifying the Studies

The Preferred Reporting Items for Systematic Review and Meta-Analysis Protocols (PRISMA) guidelines [29] were used to identify relevant studies in four databases (PsycINFO, Web of Science, Scopus, PubMed) searched on 22 December 2020. No limits were set on the year of publication (the databases went back to 1945) and the following families of keywords were used:

- relating to the neurodegenerative disease of the population considered, i.e., dementia OR Alzheimer’s disease OR mild cognitive impairment (MCI);
- in combination (AND) with horticultural therapy OR garden therapy OR healing garden OR therapeutic garden OR wander garden OR gardens for cognitive impairment.

Our electronic searches identified 480 articles, which were reduced to 347 after deduplication. The first and last authors independently read the titles and abstracts of the 347 papers and selected articles meeting our inclusion and exclusion criteria. They compared their selections and came to an agreement on the two discordant articles, ultimately identifying a total of 30 articles. One other paper would have been eligible according to our criteria, but it was not available and the authors did not respond to our request for a copy [30]. The 30 articles were read by the first author and a second expert, who independently selected 16 to be analyzed in the next stage (two of these articles were included after a discussion between the two reviewers). The following inclusion and exclusion criteria were adopted to identify the final set of 16 articles.

### 2.2. Inclusion Criteria

- Type of publication: articles published in scientific journals
- Language: English
- Research design: experimental or quasi-experimental, with transversal or longitudinal designs, and studies with a control group, OR using correlational methods that relate the time spent in the gardens with the outcomes; individual case studies
- Type of intervention: therapeutic gardening or horticultural activities in indoor and outdoor natural settings
- Population: people with MCI, PWD (Alzheimer’s disease or other types of dementia)
- Reference setting: therapeutic gardens for PWD in various residential care facilities, i.e., adult day services [31,32], dementia care units [33,34,35,36], nursing homes [32,37,38,39,40,41], long-term care settings [42], care institutions for dementia patients [43], hospitals [39,44,45], and mental health services [46].

### 2.3. Exclusion Criteria

- Books, chapters of books, doctoral theses, proceedings
- Articles based exclusively on descriptive studies, expert opinions
- Reviews and meta-analyses
- Studies on typically-functioning elderly people
- Studies involving patients with diseases other than dementia
- Studies on the effects of mere exposure to nature in various settings, rather than on therapeutic gardens or gardening.

**Figure 1:**
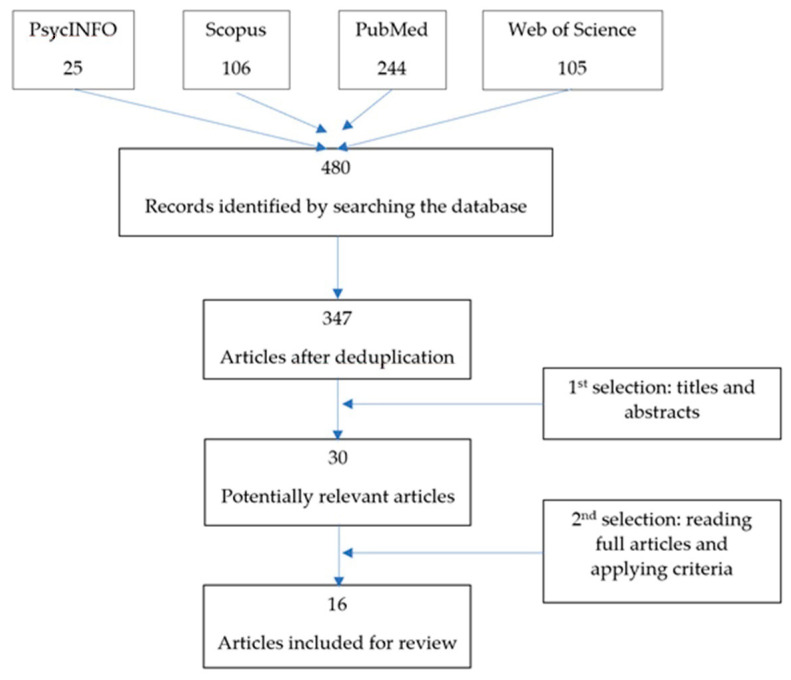
Flow diagram for article selection.

The article selection procedure is graphically illustrated in Figure 1.

## 3. Results

The main characteristics of the studies are summarized in Table 1.

Table 2 provides details of the participants in the 16 studies.

### 3.1. Characteristics of the Studies

#### 3.1.1. Types of Study, Country, Year of Publication

The methods and results of the studies are summarized Table A3 in Appendix A. Among the 16 articles selected—see Table 2—(3 of which refer to the same study [33,34,35]), there were 10 with a pre-test, post-test research design [33,34,36,37,38,40,42,43,45,46], 5 included a control group [32,34,36,37,45], 4 adopted a multiple treatment design [31,39,41,44], and 2 were studies on single cases [41,42].

The studies were conducted in the United States [31,32,33,34,35,41,42], China [37], Australia [38], England [40,46], South Korea [43], Japan [39,44], Italy [36], and France [45]. The majority were performed in the United States (n = 7) and Asia (n = 4).

The years of publication ranged from 2005 to 2020, with a greater frequency between 2017 and 2020 (n = 7), which goes to show that interest in this topic has grown in recent years. Ten articles analyzed the effects of exposure to and the use of therapeutic gardens [36,38,45], wander gardens [33,34,35], sensory gardens [41], traditional Japanese gardens [39,44], and natural gardens [40]. Six studies examined the influence of structured gardening activities [31,32,37,42,43,46].

#### 3.1.2. Characteristics of the Samples

Most of the samples consisted of less than 50 participants, except for two that included 129 [32] and 163 [36]. The participants’ ages ranged from 43 to 98, with most studies involving people over 74. All participants had been diagnosed with dementia (as required by our inclusion criteria), but differed in the type and severity of their disease, as detailed in Table 2.

#### 3.1.3. Assessment Measures

All the studies used various quantitative measures, depending on the variable of interest (as listed in Table A3). Five studies also contained qualitative observations. The quantitative tools used were: an ad hoc observational scale (the Behavioral Assessment Checklist; BAC), and the Menorah Park Engagement Scale (MPES) [47] for engagement; an ad hoc revised version of the Dementia Care Mapping (DCM) scale [48], the Apparent Affect Rating Scale (AARS) [49], and the Beck Anxiety Inventory (BAI) [50] for affect; incident reports, the Cohen-Mansfield Agitation Inventory (CMAI) [51], and the Neuropsychiatric Inventory (NPI) [52] for behavior; clinical records and other data sources for falls and medication; the CMAI [51], and the Agitated Behavior Mapping Instrument (ABMI) [53] for agitation; the Dementia Quality of Life Instrument (DEMQOL) [54], the Bradford Well-Being Profile (BWBP) [55], and the Bristol Activities of Daily Living Scale (BADLS) for quality of life [56]; the Cornell Scale of Depression in Dementia (CSDD) [57], datasheets, and the Geriatric Depression Scale (GDS) [58] for mood and depression; the Mini Mental State Examination (MMSE) [59], and the revised version of the Hasegawa Dementia Scale (HDS) [60] for cognition; a diary for sleep; physiological indicators such as heart rate and cortisol levels for stress; the Self-Consciousness Questionnaire (SCQ) [61,62,63] for self-consciousness; and the Barthel Index (BI) [64] for activities of daily living.

The gardening activities involved in the studies are described in Appendix A, Table A1 and the types of garden used in the studies are described in Appendix A, Table A2.

### 3.2. Findings in Regard to Our Research Questions

Table A3 in Appendix A summarizes the results of the studies grouped by the outcomes of interest and the measuring tools used.

Overall, of the 16 studies reviewed, 14 reported significant improvements in PWD in one or more areas following horticultural therapy or the use of a therapeutic garden. All 10 papers analyzing the effects of using or being exposed to therapeutic gardens showed improvements in one or more of the categories analyzed, including: Engagement [39,44], Behavior [33,36], Medication [33,34,36], Falls [34], Agitation [35,38,41], Quality of life [38,41], Stress [36,39,44], Depression/Mood [38,40], Cognition [36], and Self-Consciousness [45]. The 6 studies that focused on horticultural activities also reported positive findings for numerous variables: Engagement [31,32]; Affect [31,42]; Falls [42]; Sleep [43]; Agitation [43]; Cognition [43]; and Depression [42].

No improvement was found in two studies [37,46]. One involved a small sample (seven PWD in the intervention group and six in the control group) and a short period of time (6 weeks), and both these factors could have prevented any positive effects from coming to light. The other was also conducted on a small sample (initially 12, later reduced to 9) of people with early-onset dementia (age range 42–65 years), who were all very satisfied with the affective and motivational effects of their gardening experiment; the lack of any apparent improvement in their cognitive functioning was probably due to the characteristics of the sample.

The findings in the various categories analyzed are detailed below.

#### 3.2.1. Engagement

In all, four studies reported outcomes in the Engagement category.

Gigliotti and Jarrott [31] assessed engagement during gardening activities (horticultural therapy (HT) for half an hour, once a week, for 9 weeks, in groups of up to six participants) compared with traditional activities (TA: puzzles, exercises, games, crafts) in 48 PWD with moderate cognitive problems attending four adult day centers. Levels of active engagement were expected to be higher during HT than during TA, and non-engagement levels were expected to be lower.

Every 5 min, two research assistants recorded the behavior of each participant using four codes: social (verbal/non-verbal social interaction, with no other activities); gardening (planting, watering, etc.); productive (actively engaged in an activity other than gardening, such as singing, reading, etc.); and non-engaged (repetitive, self-stimulating behaviors, sleeping). The mean percentage of time engaging in gardening during the sessions was 78% (compared to 28% of productive behavior in TA; *p <* 0.001); non-engaged time was 14% during HT and 60% during TA (*p <* 0.001).

Then Jarrott and Gigliotti [32] compared the engagement of 75 PWD involved in HT with that of 54 PWD involved in TA. The study involved eight care programs, four randomly assigned to the treatment condition (HT twice a week for 6 weeks) and four to TA. Active engagement, passive engagement, self-stimulation, non-engagement, and engagement in activities other than the one presented were recorded and analyzed. Differences between the two groups, in favor of the treatment group, were significant for: active engagement (*p* < 0.01), self-stimulation (*p* < 0.01), passive engagement (*p* = 0.01), and engagement in other activities (*p* < 0.01). No significant differences emerged for non-engagement, with low rates of non-engagement in both groups.

Goto et al. [44] assessed the effects of viewing a Japanese garden (JG) on attentive behavior in 25 hospitalized patients with middle- to late-stage dementia under four within-subject conditions (6 patients participated in all conditions): an open view of the site (a terrace with plants) before the JG was built (control; Test 1); an open view of the JG (Test 2); a view of the JG through a closed door (Test 3); same as Test 3 but with a chrysanthemum scent (Test 4). During each 15-min visit, participants sat 1.5 m away from a glass door onto the garden, and their attention to the garden was recorded with a behavioral assessment checklist. The average level of attention increased significantly from Test 1 to Test 2 (*p* < 0.005). Eye movements were recorded for another group of patients with mild cognitive impairment seated with a view of the JG, and of a control view. No statistical analyses were reported, but the findings suggested that participants’ visual attention was drawn more in the direction of the JG, than towards the control view.

In a similar study conducted on PWD from different cultural backgrounds [39], differences were found between the various conditions of the JG and the control setting (*p <* 0.05, for the door both open and closed). Differences were described for other scores too, but the results were not significant.

#### 3.2.2. Affect

In the first work done by Gigliotti and Jarrott [31], the impact of gardening on affect was measured with a revised version of the DCM scale to test the hypothesis that PWD experienced a more positive emotional state during HT than during TA. Their findings supported this hypothesis (*p <* 0.001). In their subsequent study [32], however, when the AARS was used to measure affect, no significant differences emerged between the two types of activity in any of the categories considered: pleasure (*p* = 0.123), anxiety (*p* = 0.932), interest (*p* = 0.208).

In a single case study on a 76-year-old woman diagnosed with moderate dementia, Mitchell and Van Puymbroeck [42] compared her levels of anxiety and depression before and after 17 sessions of therapeutic gardening and cognitive behavioral therapy (CBT) for 40–60 min a day, 3–4 times a week for 6 weeks. Using the BAI, they found a 36% improvement in anxiety and depression from test to re-test.

#### 3.2.3. Depression/Mood

Edwards et al. [38] examined whether the construction of a therapeutic garden at a nursing home would reduce patients’ depressive symptoms. They used the Cornell Scale of Depression in Dementia (SCDD) to obtain pre- and post-treatment measures for a group of patients (n = 10). The comparison was drawn between the results obtained three months before, as opposed to three months after, the garden was built. An improvement emerged, with a 13.3% (*p* = 0.02) drop in the sample’s depression score.

The study by White et al. [40] investigated whether exposure to a renovated natural garden was associated with an improvement in mood in people diagnosed with moderate-advanced dementia. In a one-year observational study, mood was scored for 28 residents before and after they went outside, for a total of 853 observations. It emerged that their mood scores improved significantly after going outside (mean change = 0.44, *p <* 0.001). Spending more time outside was also associated with an increasingly positive mood score, but the change was not linear. Marked improvements were associated with remaining outdoors for more than 20 min, and the greatest benefit was associated with periods of 80–90 min spent outdoors.

In the single case study on a 76-year-old woman conducted by Mitchell and Van Puymbroeck [42], the effects of gardening and CBT sessions on depressive symptoms were analyzed by administering the GDS before and after the intervention. The score changed from 12 out of 15 (severe depression) before the intervention to 4 out of 15 (no depression) afterwards.

#### 3.2.4. Agitation

Five studies considered the influence of therapeutic gardens and/or gardening on agitation in PWD.

Referring to the same study as in Detweiler et al. [33,34], Murphy et al. [35] found that the more participants visited the garden the less agitated they became (*p <* 0.05). The effects were greater for PWD with initially higher levels of agitation. The impact of walking capacity was also statistically significant (*p* = 0.012), indicating that voluntary visits to the garden improve the state of agitation in PWD, but not if they have difficulty walking. This latter result was discussed in the light of inclusive design recommendations.

Luk et al. [37] assessed the effects of gardening on agitation by comparing two groups of PWD (mean MMSE score 13.4 in both groups): an experimental group (n = 7) took part in a 30-min outdoor gardening activity twice a week for 6 weeks, while the control group (n = 7) participated in other activities indoors, including origami, puzzles, painting, and collage. The Chinese version of the CMAI was administered before and after the intervention. No significant differences came to light, neither between the two groups’ total scores after the intervention (*p* = 0.116), nor within each group between before and after the treatment (experimental group: *p* = 0.115 and control group *p* = 0.249). The authors mentioned the small number of participants as a limitation of the study that might have prevented them from finding any differences.

Edwards et al. [38] studied the effects of a conservatory and a therapeutic garden (both complying with the principles of therapeutic garden design) on 10 patients (7 with Alzheimer’s disease, 2 with dementia of unspecified type, 1 with mixed dementia). Participants were assessed on agitation with the CMAI, three months before and again after the conservatory and garden were built. The results showed a 46.7% decrease in the mean agitation score (*p* < 0.001).

Similar results were reported by Lee and Kim [43], who analyzed the impact of indoor gardening on agitation levels in 23 PWD at a residential dementia care home. The study lasted 5 weeks, the first for a baseline assessment and the other 4 for the treatment (indoor gardening for an hour in the morning and an hour in the afternoon for 28 days). Agitation was measured with the modified CMAI (M-CMAI) once a day for 7 days in weeks 1 and 5. The authors found that indoor gardening reduced agitation levels, with a significant difference between the initial and final scores (*p* = 0.001).

Finally, Collins et al. [41] conducted an observational study with multiple treatments involving four women aged 77-95 (three diagnosed with dementia, one with Alzheimer’s disease) at a residential-care retirement community. The study lasted 12 weeks divided into four phases: A_1_ (2 weeks—no garden), B (4 weeks—indoor sensory garden for 30–45 min a day, 3 times a week), BC (4 weeks—outdoor sensory garden for 30–45 min a day, 3 times a week), A_2_ (2 weeks—no garden). The CMAI and ABMI were used to examine the influence of the sensory gardens on the women’s agitation levels. The fluctuations in the scores during the various phases suggested an overall improvement in agitation levels associated with both sensory gardens, and especially the one outdoors. This applied to the categories in the ABMI (non-aggressive verbal behavior, non-aggressive physical behavior, verbal aggression, physical aggression) and to the CMAI scores.

#### 3.2.5. Quality of Life/Wellbeing

Three studies examined quality of life and degree of wellbeing.

The above-described study by Collins et al. [41] analyzed the influence of exposure to sensory gardens on wellbeing using the DEMQOL and DEMQOL Proxy. The results were encouraging, showing an improvement after exposure to the sensory gardens, especially the one indoors.

In the study by Edwards et al. [38] on a sample of 10 PWD living in a nursing home, DEMQOL and DEMQOL Proxy scores collected three months before a therapeutic garden was built were compared with those obtained three months afterwards. The study found an improvement in the quality of life for these PWD, with the average score increasing by 12.8% (*p* < 0.001).

Along the same lines, Hewitt et al. [46] published the results of a one-year preliminary study (run from 12 May 2009 to 10 May 2010) on a group of people with young-onset dementia (n = 12, mean age 58.6). They attended 46 sessions of structured gardening activities (an hour a week for a year, plus guided group meetings beforehand and afterwards, for a total of 2 h of activity each time). Their degree of wellbeing, assessed with both quantitative (Bradford Well-Being Profile) and qualitative measures, showed a stable trend with no significant differences, neither between sessions 1 and 21 (*p* = 0.21), nor between sessions 22 and 46 (*p* = 0.425).

#### 3.2.6. Self-Consciousness

Self-consciousness was analyzed in a study by Gueib et al. [45], using the Self-Consciousness Questionnaire (SCQ), which consists of 14 items covering seven dimensions: Anosognosia, Prospective memory, Capacity for introspection, Self-assessment of affective state, Moral judgments, Personal identity, and Body representation. The study aimed to measure the effects of a therapeutic garden on hospitalized patients with Alzheimer’s disease and related disorders. The garden included features related to nature, art, and the regional culture (Art, Memory, and Life garden). It was used by the experimental group (n = 16) for a minimum cumulative period of 12 h over 2 weeks. Each participant in the experimental group could spontaneously choose to spend time in the garden alone, with family members or with health personnel, engaging in activities or simply enjoying the place, sitting, or walking about. The control group (n=18) consisted of patients who deliberately never entered the garden for a period of two weeks. From T0 (before using the garden) to T1 (after the two weeks) there was an increase in the overall SCQ score for the group using the garden due to a significant improvement in body representation (*p* = 0.02), whereas this score decreased significantly in the control group (*p* = 0.03) during the same period, due to an increase in anosognosia. No differences emerged for any of the other variables—i.e., cognitive level (MMSE), executive functions (Frontal Assessment Battery), behavioral and psychological disorders (Neuropsychiatric Inventory for Nursing Homes; NPI-NH), or depressive state (Mini Geriatric Depression Scale)—apart from a significant decrease in total NPI-NH score between assessments at T0 and T1, in both groups.

#### 3.2.7. Sleep

The only study that considered the therapeutic effects of gardens and gardening on sleep was conducted by Lee and Kim [43]. The following components of sleep were measured: frequency and duration of nocturnal awakenings, frequency and duration of naps during the day, duration and effectiveness of nocturnal sleep, time of beginning night sleep, time of awakening in the morning, time of total sleep. The recordings were entered in a diary (24 h a day by one of the research assistants) before and after the treatment (gardening sessions) and then compared. Significant improvements emerged, with a decrease in the frequency and duration of nocturnal awakenings (frequency: *p* = 0.002, duration: *p* = 0.011) and naps (frequency: *p* < 0.001, duration: *p* < 0.001), and an increase in nocturnal sleep time (*p* = 0.002) and efficacy (*p* = 0.006). No changes emerged for the time of sleep onset (*p* = 0.134), the time of awakening in the morning (*p* = 0.114) or the total sleep time (*p* = 0.976).

#### 3.2.8. Stress

Three studies investigated stress. Goto et al. [39,44] measured heart rate (beats per minute, BPM) as a physiological stress indicator in participants responding to seeing a Japanese garden. They compared BPT measures at T1 (before the garden was built), T2 (on viewing the garden from inside a room with the door open), T3 (as in T2 but with the door closed), and T4 (as in T3 but with the scent of chrysanthemums). There was a significant drop in BPT from T1 to T2 (*p <* 0.05), while heart rates at T3 and T4 were between those at T1 and T2, with fewer BPT at T4 than at T3, confirming that closing the door reduced the benefits by comparison with having the door open.

In the study by Pedrinolla et al. [36], stress was measured in terms of cortisol levels (obtained by sampling saliva) and systolic and diastolic blood pressure. An experimental group that experienced an indoor therapeutic garden was compared with a control group that did not, but spent time elsewhere in the building. Significant differences emerged in favor of the control group, with differences between the two groups in the range of −6.4 to −2.1 Nmol/L (*p* < 0.001) for cortisol levels measured at various times of day, −2.6 mm Hg (*p* < 0.001) for systolic blood pressure, and −2.6 mm Hg (*p* < 0.001) for diastolic blood pressure.

#### 3.2.9. Cognition

Three studies reported changes in the cognitive functioning of PWD after experiencing therapeutic gardens or gardening [36,43,46].

Lee and Kim [43] exposed a group of 23 people with moderate-severe dementia to an indoor gardening treatment for an hour a day for 28 days. The participants’ cognitive level was measured with the Revised Hasegawa Dementia Scale (HDS-R) in the first and fifth weeks. The HDS-R consists of 5 subscales for assessing orientation, memory, calculation, attention, and semantic fluency. The difference between the initial and final scores was statistically significant (*p* < 0.001), supporting a general improvement following the gardening activity.

Hewitt et al. [46] used the MMSE as a measure of cognitive functioning in their sample of people with early-onset dementia (n = 12), tested at the baseline and again at 6 months and one year (at the end of 46 structured gardening sessions). The results revealed no significant difference after 6 months compared with the baseline (NS) and a significantly worse cognitive functioning after a year (*p* = 0.012).

The MMSE was also used in the study by Pedrinolla et al. [36], who measured cognitive functioning over a period of 6 months in an experimental group of PWD (n = 82) who had access to an indoor therapeutic garden, comparing them with a group of PWD (n = 81) who did not. The difference between each group’s initial and final MMSE scores showed a significant change of 1.8 points in the control group (*p* < 0.001).

#### 3.2.10. Behavior

Detweiler et al. [33] conducted an observational study covering two consecutive years on a group of 34 male residents of a dementia care unit to assess the influence of providing a wander garden at the facility on their inappropriate behavior. The authors used a short version of the CMAI, which considers 14 levels of aggressive behavior on a 5-point scale, recording any such episodes for each participant. The records were kept during the year before the wander garden was installed and during the year afterwards. The CMAI scores decreased from 21.88 to 18.9 (Cohen’s *d* = 0.64). Significant correlations were found between the final CMAI scores and the total number of days spent in the garden (more time spent in the garden correlating with lower CMAI scores; *p* < 0.05), and between the initial and final CMAI scores (*p* < 0.01). Lower CMAI scores emerged for the group visiting the garden more often, while there was no correlation between the final CMAI scores and the number of incidents reported. Findings regarding participants’ inappropriate behavior before and after the garden was built generated conflicting results, however: there was no difference between pre- and post-test for inappropriate behavior classified as having a severity of 1–3 (mild-moderate), while there was a significant difference for level 4 (the most severe), with an unexpected increase in such behavior during the second year. Discussing this result, the authors suggest that it could be explained by participants’ individual characteristics, and they emphasized the lack of a control group as a limitation of their study. Other issues mentioned by the authors concern the weather, which was too cold from mid-October to mid-March for residents to be allowed to visit the garden, and their use of the garden was limited from June to August because it was too hot and there was a shortage of shady areas. Some residents’ inappropriate behavior could also have been linked to environmental factors, such as architectural barriers that limited their use of the garden. For example, in June the doors to the garden were closed at dusk, but the outdoor lights remained on, so residents tried to go there, and found their way blocked.

The study by Pedrinolla et al. [36] also examined behavior, finding an improvement in behavioral symptoms (measured with the NPI) in an experimental group that was given access to an indoor garden compared with a control group that was not (*p* < 0.001).

#### 3.2.11. Falls

Detweiler et al. [34] recorded falls before and after a garden was added at a residential care facility, and looked for differences in the number falls between groups making more or less use of the garden. The number of falls decreased from 288 to 200 in the year after the garden was opened. The group making more use of the garden showed a 38.7% reduction in the number of falls from before to after the intervention, while for the group making less use of the garden the improvement was only 7.9%, and the difference between the two groups was significant (*p* < 0.05). That said, the former (high use) group had many more falls at the baseline than the latter (low use) group (282 vs. 97, respectively), and this difference between the two groups persisted in the second year. As for fall severity scores (based on the Institutional Fall Committee ratings of each reported fall), the monthly mean score decreased from 1.15 to 0.81 in the sample as a whole. Grade 1 falls (the least severe, causing no injury) were the most common in both years, and showed a marginally significant decrease in number (0.05 < *p* <0.1), while the differences for the more severe falls were never significant.

When the two groups were compared on their total fall severity scores, a clearer pattern emerged: there was a reduction of 36.5% in the group making more use of the garden as opposed to 9.3% in the group using the garden less (the difference was significant, *p* < 0.05). Finally, when the correlation between severity of falls and medication administered was examined, a positive correlation emerged in both years for: primary antidepressants (baseline year: *p* < 0.001; observation year: *p* < 0.01) (fewer antidepressants being associated with fewer falls) and anxiolytics (baseline year: *p* < 0.01; observation year: *p* < 0.05) (fewer anxiolytics again being associated with fewer falls).

In the single case study on the 76-year-old woman described by Mitchell and Van Puymbroeck [42], the number of falls before and after a 6-week therapeutic gardening activity decreased significantly from seven recorded three months before the treatment to none afterwards.

#### 3.2.12. Activities of Daily Living

The report from Pedrinolla et al. [36] describes the only study in the present review to have examined activities of daily living (using the Barthel Index), which detected no differences between the experimental and control groups.

#### 3.2.13. Medication

In the same study by Pedrinolla et al. [36], the only difference in the amount of medication (i.e., drug dosage) administered to the experimental and control groups concerned a lower dosage of quetiapine (an antipsychotic) for the former than for the latter: −150 mg (*p* < 0.001).

Detweiler et al. [33] explored the use of medication on an ‘as needed’ basis (*pro re nata* (PRN)), comparing the number of administrations to 34 male PWD a year before and a year after a therapeutic garden was built at their residential care home. There was a significant correlation between the medication administered initially and in the second year (*p* < 0.01), and the percentage of participants not requesting any medication rose from 35.3% in the first year (before the garden) to 55.9% in the second (after the garden was installed).

A year later, the same researchers [34] published another article concerning the use of medication, with data relating to a subsample (n = 28) from the previous study. Here again, they compared two groups making more (n = 14) or less (n = 14) use of the garden, and examined the use of primary and secondary antidepressants, antipsychotics, anxiolytics and hypnotics, comparing the year without the garden with the subsequent year with the garden. There were no significant differences between the groups making more or less use of garden in terms of their use of primary antidepressants (*p* > 9) or anxiolytics (*p* > 0.1), while the former group needed fewer secondary antidepressants (*p* < 0.005) and antipsychotics (regardless of whether the patients were on a high, intermediate or low dosage) (*p* < 0.001). The only difference in favor of the group making less use of the garden concerned the intake of hypnotics, which they needed less often than the group making more use of the garden (*p* < 0.001). Comparing the amount of medication administered before versus after the garden was built, there was a significant difference in the use of primary antidepressants (*p* < 0.001), which increased by 125% for patients on a high dosage, and by 83.3% for those on a low dosage, while it decreased by 34.2% for those on an intermediate dosage. A small number of patients who had been prescribed a scheduled secondary antidepressant had all their dosages reduced during the year of observation (*p* < 0.001). The use of antipsychotic medication also decreased from the baseline during the year of observation (*p <* 0.001) by 75% for patients taking a high dosage, and by a statistically insignificant percentage for the patients on low and intermediate dosages. The number of patients not using any antipsychotics increased by 21% in the second year. No significant differences came to light for anxiolytics or hypnotics.

## 4. Discussion

### 4.1. Research Questions and Related Answers

Analyzing the main findings of the studies reviewed gave us an opportunity to address our research questions.

The first concerned the efficacy of therapeutic gardening (How effective are therapeutic gardens for PWD?). Overall, the findings support the existence of positive effects extending to multiple areas. The studies on therapeutic gardens, wander gardens, sensory gardens, Japanese gardens, and a renovated natural garden all showed improvements in one or more areas: Engagement [39,44], Behavior [33,36], Medication [33,34,36], Falls [34], Agitation [35,38,41], Quality of life [38,41], Stress [36,39,44], Depression/Mood [38,40], Cognition [36], and Self-Consciousness [45]. These outcomes point to the importance of including green areas and specific gardening activities in residential facilities for PWD as a means to enhance their quality of life.

Regarding the second question (Which domains—behavioral, cognitive, mood, sleep, physiological, etc.—are the most affected?), the domains most affected by exposure to or use of garden, or involvement in structured horticultural activities were behavior, with a reduction in aggressive behaviors (2 out of 2), engagement (4 out of 4), agitation (4 out of 5), and falls (2 out of 2). Mood also improved, with lower levels of depression (3 out of 3) and stress (3 out of 3), and enhancements in positive affect (2 out of 3). Another important effect observed was a reduced use of medication (3 out of 3), probably due to improvements in the behavioral area. Other specific variables were positively affected, from cognition (2 out of 3) to self-consciousness (1 in 1). Finally, there was evidence of a general improvement in quality of life and wellbeing (2 out of 3), and quality of sleep (1 out of 1).

Less can be said regarding the third question (Which garden design features have the greatest effects?) because the findings concerning this issue were very limited in the studies selected. None of the studies drew any direct comparisons between different types of garden—except for Collins et al. [41], who compared an indoor with an outdoor garden, finding the latter more beneficial. Design features were described in more or less detail, but none of the studies specifically examined the environmental variables to test their influence on PWD.

As for the fourth question about which activities undertaken in the garden are the most effective (structured activities like gardening or recreational activities such as doing physical exercises, spending time in the garden, or walking), none of the studies compared the specific effects of different activities in the garden. The types of activity were sometimes not stated, or they varied within the same study from mere exposure to a garden to horticultural activities. In some cases, participants experienced the garden along with activities involving art, bricolage, or psychotherapy. The lack of any direct comparisons between the various activities makes it impossible to establish whether a particular activity prompted the effects described.

Overall, therapeutic gardening would seem particularly suited to people who are aging in general, and to PWD in particular. As several contributions have emphasized [65], depression in aging is often associated with high levels of anxiety, somatic symptoms (generalized pain, back pain, sleep disturbances, reduced appetite, weight loss), and cognitive loss—the latter being both a cause and an effect of depressive mood. Old people can also suffer from subclinical mood disorders (such as demoralization) [66,67], which can have a strong impact on their quality of life, even to the point of making them suicidal [67,68,69]. Providing safe ways for them to spend time in contact with nature and restorative gardening activities is a relatively easy way to prevent or contain these problems.

The importance of dedicating a specific space to the rehabilitation of PWD is particularly important because they are extremely vulnerable, living in a condition where physical illness is compounded by moral and physical pain [65,66,67].

The present work confirms that therapeutic gardening can be helpful not only in “pure” forms of Alzheimer’s disease (only a small number of the studies reviewed considered this type of patient), but also in the more common mixed forms of dementia [70,71].

### 4.2. Limitations and Future Developments

The studies reviewed here leave a number of questions unanswered that should be addressed in future research. For example, it would be useful to examine which activities are the most effective (and their timing), and the most suitable garden design as a function of the severity and type of people’s dementia. None of the studies considered here specifically compared the efficacy of different types of activity (which ranged from mere exposure to a green area to gardening activities or other outdoor activities in a green space), and only one specifically addressed the effects in relation to the amount of time spent in the garden.

More information is needed on the possible interactions between different categories of patients and gardening, as only two studies tested whether the effects of using a garden differed depending on the severity and type of dementia.

More attention should be paid to garden design factors as well (such as the types of plant, the general layout, any water or ornamental features, familiar objects, pictures of familiar places, etc.), and to other design variables, such as the view of the garden from windows or glass doors, how easy it is to access, the negative effect of barriers such as heavy doors, and so on.

It would also be interesting to consider other categories of elderly in the same residential settings who might benefit from exposure to garden, such as staff and family caregivers. Promisingly, two studies reported on the impressions of family members and caregivers as regards the effects of a therapeutic garden and structured gardening activities on their own levels of wellbeing, as well as those of the PWD.

From a methodological point of view, studies on larger samples with more controlled designs (use of control groups and randomization) are to be recommended.

## 5. Conclusions

In the last decade, there has been growing interest in therapeutic gardens and gardening therapy for PWD. Studies on their use have been inspiring and have considerable applicative value. The efficacy of therapeutic gardens and gardening in improving various behavioral, affective, and cognitive areas seems to emerge from all the studies reviewed here. Taken together, our findings directly support the efficacy of therapeutic gardening in improving different areas of wellbeing in PWD. They also indirectly support theories on environmental restoration and the beneficial effects of contact with nature for human wellbeing [2,4].

Though more research would be needed to clarify the above-mentioned limitations, current and future research on therapeutic gardens can usefully contribute to our understanding of how humans interact with the natural world, and how to ameliorate the life of PWD and their formal and informal caregivers.

## Figures and Tables

**Table 1:** Main characteristics of the studies.

Characteristics		Studies Selected (As Numbered in Reference List)			
Number of participants	1 < N < 10	[38,41,42]			
	11 < N < 20	[37,46]			
	21 < N < 30	[34,40,43,44]			
	31 < N < 40	[33,35,39,45]			
	41 < N < 50	[31]			
	51 < N < 100	/			
	101 < N < 150	[32]			
	151 < N < 200	[36]			
Study design		**Pre-test—Post-test**	**Longitudinal**	Multiple treatment *	Comparative **
	No control group [31,33,35,38,39,40,43,44,46]	[33,38,40,43,46]	[35]	[31,39,44]	
	With control group [32,34,36,37,45]	[34,36,37,45]			[32]
	Single case [41,42]	[42]		[41]	
Data collection methods	Family report	[33,36,38,46]			
	Staff report	[33,38,39,40,41,43,44,46]			
	Researcher report—direct observation	[31,32,33,34,35,36,37,39,42,43,44]			
	Task/test administered to participants	[36,37,38,41,42,43,45,46]			
Measurements	Quantitative	[32,34,35,36,37,39,40,41,42,43,44,45]			
	Mixed ***	[31,33,38,46]			
Country of the study	USA [31,32,33,34,35,41,42]; CHINA [37]; AUSTRALIA [38]; UK [40,46]; SOUTH KOREA [43] JAPAN [39,44]; ITALY [36]; FRANCE [45]				

Notes: * a baseline followed by separate phases in which different treatments were introduced; ** comparing groups given different treatments; *** quantitative and qualitative.

**Table 2:** Summary of the studies reviewed (n = 16).

Study	Country	Sample	Sex	Type of Dementia	Stage of Dementia	Age of Participants
[31]	USA	48	26 M/22 F	unspecified	MMSE: *M* = 13.07	46–98 (*M* = 80)
[33]	USA	34 (final sample 29)	34 M	unspecified	n.a.	74–92 (*M* = 80.71)
[43]	SOUTH KOREA	23	/	unspecified	mild or severe	/
[34]	USA	28	28 M	unspecified	n.a.	74–92 (*M* = 80.5)
[32]	USA	129	53% F	unspecified	MMSE: *M* = 9.62	*M* = 80
[35]	USA	34	34 M	unspecified	n.a.	74–92 (M *=* 80.71)
[37]	CHINA	14	1 M/13 F	unspecified	MMSE: *M* = 13.4	*M* = 84.9
[38]	AUSTRALIA	10	1 M/9 F	7 Alzheimer’s disease, 2 unspecified, 1 mixed	4 severe, 3 moderate, 3 mild	79–90
[46]	UK	12	4 M/8 F	Young-onset dementia: 9 Alzheimer’s disease, 1 frontotemporal, 1 mixed Alzheimer’s and vascular, 1 dementia with Lewy bodies	MMSE: *M* = 17, range = 8;28	43–65 (*M* = 58.6)
[44]	JAPAN	25 (6 in all conditions)	n.a.	unspecified	middle-late MMSE: *M* = 10	*M* = 91
[39]	JAPAN	16 + 16 (6 in all conditions)	n.a.	unspecified	8 severe, 14 moderate, 8 mild (no data on 2) MMSE: *M* = 12	*M* = 91
[40]	UK	28	n.a.	unspecified	middle-late	/
[42]	USA	1	1 F	unspecified	moderate	76
[36]	ITALY	163	42 M/121 F	Alzheimer’s disease	MMSE: M *=* 13	*M* = 77
[41]	USA	4	4 F	3 unspecified, 1 Alzheimer’s disease	n.a.	77, 95, 92, 95
[45]	FRANCE	34	13 M/21 F	Alzheimer’s disease and related disorders	MMSE: control group *M* = 12.4 experimental group *M* = 10.2	*M* = 82

## Appendix A

**Table A1:** Horticultural/gardening activities presented in the studies.

Study	Activity	Description of Activity
[31]	Horticultural therapy, 30 min per session, once a week for 9 weeks	The time of year when the activities were undertaken is not stated, but each group had horticulture therapy in more than one setting, depending on the summer heat (indoors, in a screened-in porch, in an outdoor area with raised beds). Two horticulture therapists with experience of working with older adults planned the sessions, some involving teamwork, others conducted individually in parallel activities. The care home staff were invited to help with the horticultural activity (during the sessions with individuals who needed one-to-one attention). Facilitators presented the activity, encouraged social interaction and reminiscencing with questions about participants’ social stories and past experiences of gardening, farming, cooking, and other related topics. Before each activity, the facilitators prepared the material for each participant, involving them in the choice of plants and containers. The activities were personalized. For example, individuals with a tendency to wander were instructed to fill watering cans for others who had more mobility issues, and those who were bothered by dirt were given gloves. Adapted gardening tools were used.
[43]	Indoor gardening, 1 h per session, twice a day for 4 weeks	The time of year when the activities were undertaken is not stated. Each individual was assisted by three registered nurses in the choice of their preferred plant and a name to give their container. The containers were located in the day room at the care home and could be accessed all day. Activities included: picking seeds, filling containers, planting roots or seeds, touching, watering, organizing containers, cleaning floors, harvesting, cutting, and washing. Participants had to make multiple trips to fill small watering cans and wash dirty cloths. They were encouraged to look at and touch their plants whenever they wished and, once harvested, the products were served as a side dish with their meals. The plants chosen were filipendula and soybean sprouts as they are familiar, affordable, edible, easy to grow and bloom, and require little space.
[32]	Horticultural therapy, 50 min per session, twice a week for 6 weeks	The time of year when the activities were undertaken is not stated. Two facilitators developed the activities, chosen for their simplicity, cost and versatility. When a group exceeded eight participants it was split into two to provide step-by-step assistance and a constant supply of materials to participants. Activities ranged from sowing to training topiaries to craft activities that included horticultural materials and themes. Participants were encouraged to interact and remember through questions about gardening and cooking. The activities depended on the plants in season. Participation was voluntary and proposed to elderly people who were told that the activity would involve gardening.
[37]	Horticultural therapy, 30 min per session, twice a week for 6 weeks	The time of year when the activities were undertaken and the type of trainer are not stated. Each session had a different theme (sowing, fertilizing, planting, caring for flowers).
[46]	Structured gardening program, 1 h a week, 46 sessions	From May 2009 to May 2010 Each session was arranged to include first some time to plan the activity and socialize, then an hour of gardening followed by a moment of reflection and discussion on the activities. There were 6 occupational therapists, a horticultural therapist, a psychologist, a volunteer, and a support worker at each session. A flexible and adaptive approach, based on positive reinforcement, was used. Activities: digging and planting spring flowering bulbs in the flowerbeds, removing leaves, sensory activities. Options were given where possible so that participants could have choice and autonomy. After each session, a diary was updated with photos and written information that participants used to communicate with their relatives.
[42]	Therapeutic gardening and cognitive behavioral therapy, 40/60-min sessions, 3–4 times a week, over 6 weeks	The time of year when the activities were undertaken is not stated. A weekly calendar indicated the day, time, and type of activity for the day to encourage a sense of autonomy and competence, and to promote a sense of readiness for the therapist’s arrival. It was sometimes necessary to encourage PWD to take part in the activity, to support their efforts in the garden as much as possible, involve them in discussions about their own care, and encourage their active participation, giving voice to the needs related to horticulture and other issues. Simple tasks: picking seeds, planting, taking care of the garden. Patients were encouraged to independently choose 8 flowers and plants to put in a flower bed. They chose the better-known ones and the therapist encouraged their memories with questions about their history and their family.

**Table A2:** Gardens presented in the studies.

Study	Type of Garden	Description of the Garden
[33] [34] [35]	Wander garden	The garden could be seen in its entirety from a large floor-to-ceiling wide window in the dining room, which was also used as an activity room. Two doors, one on each side, led to a walkway that went around the perimeter of the garden. Two of the three outer walls of the walkway had large windows and there were three doors for exiting the garden. These doors had the standard electronic constraints to prevent escape. The third outer wall had a small window with panes above eye level for providing natural light without offering a view of the outside environment. The doors to the wander garden were usually open after breakfast and closed after dinner, even when the weather was unfavorable. Activities comprised daily garden viewing, garden walks, and garden activities when the weather allowed. [32] The garden was used less in the winter months but residents of the nursing home could see it through the dining room window. From mid-October to mid-March the garden remained closed due to the cold. It was also closed for 2 weeks in April for administrative reasons. The doors to the garden were closed at 4:30 pm in April and May, and at 8 pm in June. In July and August it was too hot to go out before 4–5 pm. [36] Visits to the garden were mainly between August and October, and increased over this time, then decreased between December and February. They increased again between March and April, leveling off until July, but no longer reached the numbers of the first months of opening.
[38]	Therapeutic garden	The garden was used in Spring. Elements in the garden included memory boxes, a tinka car, a platform overlooking the Australian countryside, a woodpile, an aviary, a quiet area with water features, and flower beds where residents can dig and pick products.
[39] [44]	Japanese garden	Abstract naturalistic garden with plants, stones, stepping stones, bamboo fences, stone basins, stone lanterns. [44] TEST 1: 16 March–4 April (participants were seated so as to view the unreconstructed, natural area from an open door. TEST 2: 22 June–10 July (same situation as TEST 1 but viewing a Japanese garden); TEST 3: from 19 to 25 October (the summer months were skipped due to the heat; same situation as TEST 2 but with the door closed); TEST 4, a week after TEST3 (same situation as TEST 3 but with chrysanthemum scent). [39] TEST 1: 1–15 April (before the construction of the gardens); TEST 2: 1–15 June (after the construction of the gardens, viewing with the door open); TEST 3: 19–25 October (summer skipped due to the heat; viewing the gardens with the door closed). The tests took place from 9:30 to 12:30, and from 13:00 to 16:30. While participants were viewing the gardens, the staff recorded heart rate and behavior, and did not encourage discussion, but only answered the participants’ questions.
[40]	Nature garden	Observations throughout the year except January, peaking from May to September. Renovated space with fruit trees and vegetable beds, both passive and active spaces, following previously-established guidelines.
[36]	Indoor therapeutic garden	The sessions took place in the morning between 9:00 and 14:00, in groups of about 20 participants, from June 2015 to June 2016. The garden of the care home (located in the city center) included a large, purpose-built area covering 300 square meters with non-toxic plants that promote olfactory, visual, and tactile stimuli. The trail was endless and encouraged movement and contact with the plants. The entrance gave direct access to the trail, built with smooth, non-slip material. Inside the garden there were two areas for sitting and socializing, and a fountain with running water. The garden walls had large glass windows, covered with a safety film, affording a direct view from the inside. Temperature and humidity were kept constant. Garden plants: ficus benjamina, croton variegatum, aglaonema commutatum, spathiphyllum wallisii, anthurium andreanum, dracena marginata, variegated ground cover scindapsus, feijoa sellowiana, shefflera act., trinette, rosmarinus officinalis, jasminea, dwarf officinalis, white heavenly muse. Patients were accompanied by the session managers to the therapeutic garden where they strolled and were encouraged to touch the plants and flowers.
[41]	Indoor sensory garden	The time of year was not stated. The garden was placed in the dining room on the first floor, on a dining table, set apart from other activities in the room by a curtain.
	Outside sensory garden	The time of year was not stated, but the excessively cold climate limited participants’ opportunities to experience the outdoor garden. Windows in the walls of the dining room on the first floor let in natural light, enabling residents to view nature. Participants were encouraged by the researchers to interact with all the plants. They were asked what the plants reminded them of, involving all the senses—taste, touch, scent. Plants: Coriandrum sativum, Lactuca sativa “Simpson Elite”, Hosta “Patriot”, Rosemary, Brassica juncea “Red Giant”, Chrysanthemum, Dracaena.
[45]	Art memory life garden	The time of year was not stated. The garden contained artistic, natural and cultural features. It was 4000 square meters in size, accessible directly from the day room at the Cognitive Behavioral Unit. It was surrounded by buildings on three sides and it had an opening overlooking the city on the fourth. Access was reserved for residents and visitors to the center. Climate was continental, with cold winters and frequent rains. The garden faced south and the building was to the north, with both sunny and shaded areas thanks to the presence of plants and numerous old trees. Residents could use it as they wished, in the utmost autonomy, to contemplate the plants and artworks, use the benches, and have conversations. They could put their things on the tables, and find magazines. There were drinks available. The flowers could be touched and picked. The sculptures could be touched and were designed for tactile exploration. Residents could receive their loved ones of all ages in the garden. There were planters with small strawberry plants, herbs, and fragrant plants, and the residents could watch and water them, and taste the fruits. They could also look at the Galileo thermometer and the insect cages. They received friends and relatives with no time constraints or fixed schedules, and went to the garden with them as often as they wished. If small children were visiting, it was preferable to receive them in the garden. Residents could have meals or snacks in the garden too whenever they wished. The general criteria for creating a therapeutic garden had been adopted, including: avoiding glare, and sudden changes of lighting between indoor and outdoor spaces; hygiene and accessibility (no stagnant water); simple and clear layout to facilitate orientation; private and shared spaces, avoiding large spaces and stressful elements; a view from inside the garden; surveillance by staff; a view of the city; furniture adapted to residents’ physical needs; water features. Symbolic elements were linked to the local culture. Plants: strawberries and aromatic plants, old trees, non-toxic plants, flowers with bright colors.

**Table A3:** Methods and results of the studies.

Study	Study Design	Study Duration	Intervention/ Exposure	Control	Outcomes	Measures	Results
[31] Gigliotti and Jarrott (2005)	Multiple treatment	9 weeks	Horticultural therapy (HT), 30 min per session, once a week, 9 weeks	Same group: traditional activities (TA) (exercise, crafts, games, puzzles)	Engagement	Ad hoc observational tool	HT > TA (*t* = 13.47, *p* < 0.001)
					Affect	Ad hoc observational tool based on DCM	HT > TA (*t* = 5.15, *p* < 0.001)
[33] Detweiler et al. (2008)	Pre-test, post-test	2 years	Use of a wander garden after construction	No control group	Behavior	CMAI short form	T1 > T0 (final CMAI score with total days in the garden (r = −0.388, *p* < 0.05)
						Incident reports	No difference
					Medication	Pro re nata (PRN)	T1 > T0 (total year PRNs with total observation year PRNs baseline (r = 0.585, *p* < 0.01)
[43] Lee and Kim (2008)	Pre-test, post-test	5 weeks	Indoor gardening, 1-h sessions, twice a day, for 4 weeks	No control group	Sleep	Sleep diaries	T1 > T0 on: WASO frequency (*t* = 3.568, *p* = 0.002) and WASO duration (*t* = 2.781, *p* = 0.011); Nap frequency (*t* = 6.480, *p* < 0.001) and duration (*t* = 7.933, *p* < 0.001); NST: (*t* = -3.493, *p* = 0.002); NSE: (t = −3.048, *p* = 0.006); No difference in T1 on: Sleep onset (*t* = 1.555, *p =* 0.134); Wake up time (*t* = −1.646, *p* = 0.114); TST (*t* = −0.030, *p* = 0.976)
					Agitation	M-CMAI	T1 > T0 (*t* = −4.002, *p* = 0.001)
					Cognition	HDS-R	T1 > T0 (*t* = −12.044, *p* < 0.001)
[34] Detweiler et al. (2009)	Pre-test, post-test; single blind	2 years	Use of a wander garden after construction: HUG (high use of garden) N = 14	LUG (low use of garden) N = 14	Medications	Dosage	Likelihood ratio test: T1 > T0 in both groups Primary antidepressant (*χ*^2^ = 28.377, 3 *df, p* < 0.001), Secondary antidepressant (*χ*^2^ = 16.152, 2 *df, p* < 0.001), Antipsychotics (*χ*^2^ = 24.923, 3 *df, p* < 0.001), Hypnotics (*χ*^2^ = 5.700, 2 *df,* 0.05 < *p* < 0.1); HUG > LUG on secondary antidepressant (*χ*^2^ = 9.689, 1 *df, p* < 0.005), Antipsychotic s(*χ*^2^ = 22.618, 3 *df, p* < 0.001); LUG < HUG on Hypnotics (*χ*^2^ = 27.879, 2 *df, p* < 0.001); No difference in T1 in both groups on Anxiolytics (*χ*^2^ = 1.032, 1 *df, p >* 0.25)
					Falls	Number Severity	Total number of falls (both groups) decreases in T1 from 288 to 200; Likelihood ratio test: HUG > LUG on number (*χ*^2^ = 4.1304, 1 *df, p* < 0.05) and on severity (*χ*^2^ = 4.1298, 1 *df, p* < 0.05) also among merry walker users and wheelchair users (*χ*^2^ = 16.5296, 1 *df, p* < 0.001)
[32] Jarrott and Gigliotti (2010)	Comparative RCT	6 weeks	Horticultural therapy (HT), 50 min per session, twice a week, 6 weeks N = 75	Traditional activities (TA) N = 54	Affect	AARS (pleasure, anxiety/sadness, interest, no anger)	Wilcoxon–Mann–Whitney *U* test No difference between HT and TA: pleasure (*z* = −1.544, *p* = 0.123); anxiety (*z* = −0.086, *p =* 0.932); interest (*z* = −1.26, *p* = 0.208)
					Engagement	MPES	Wilcoxon–Mann–Whitney *U* test HT > TA on: AE (*z* = −2.90, *p* < 0.001); SE (*z* = −4.60, *p* < 0.001); PE (*z* = −2.72, *p* < 0.01); OE (*z* = 3.47, *p* < 0.001); no difference between HT and TA on NE (*z* = −1.45, *p* < 0.15)
[35] Murphy et al. (2010)	Pre-test post-test;	1 year	Use of a wander garden after construction	No control group	Agitation	Short form CMAI	A Hierarchical Linear Modeling T1 > T0 (*t* = −2.702; *p* < 0.05)
[37] Luk et al. (2011)	Pre-test, post-test; RCT; single blind	6 weeks	Horticultural therapy (HT), 30 min per session, twice a week, 6 weeks N = 7	Other activities (OA) (origami, puzzles, drawing, collage) N = 7	Agitation	C-CMAI	No difference in T1 between HT and OA (HT: *p* = 0.115; OA: *p* = 0.249)
[38] Edwards et al. (2013)	Pre-test, post-test	6 months	Use of a therapeutic garden after reconstruction	No control group	Quality of life	DEMQOL	T1 > T0 (*t* = 4.57, 9 *df*, *p* < 0.001)
					Agitation	CMAI	T1 > T0 (*t* = 7.48, 9 *df p* < 0.001)
					Depression	SCDD	T1 > T0 (*t* = 2.4, 9 *df p* < 0.02)
[46] Hewitt et al. (2013)	Pre-test, post-test	1 year	Structured gardening program 1 h a week, 46 sessions	No control group	Wellbeing	Bradford Well-Being Profile	No difference in T1: sessions 1–21 (*t* = 1.43, *p* = 0.21); sessions 22–46 (*t* = 0.88, *p* = 0.425)
					Cognition	MMSE	T1 < T0 (*t* = 3.88, *p* = 0.012)
[44] Goto et al. (2017)	Multiple treatment	7 weeks	Exposure to a Japanese garden	4 exposure tests: standard garden Test1; Japanese garden with open door Test2; Japanese garden with closed door Test3, Japanese garden with closed door plus scent Test4	Stress	Fingertip heart rate monitor	*t* test (*p* < 0.05) Test2, Test3 and Test4 > Test1; Test2 and Test4 > Test3
					Engagement	Behavioral assessment checklist	Test2, Test3 and Test4 > Test1 in attention; T2 > T1 (*p* < 0.005); Test2 > Test3 and Test4; Test4 > Test3
[39] Goto et al. (2018)	Multiple treatment	6 weeks	Exposure to two Japanese gardens: one in hospital garden and one on terrace	3 exposure tests: standard gardens Test1; Japanese garden with open door Test2; Japanese garden with closed door Test3	Stress	Fingertip heart rate monitor	Wilcoxon test and Bonferroni post-test (*p* < 0.05) Test2 > Test1 in both gardens
					Engagement	Behavioral assessment checklist	Wilcoxon test (*p* < 0.05) Test2 > Test1 on responses to gardens and on responses to caregivers in both gardens; Test2 > Test1 on memory recall in both gardens; Test3 < Test2 on positive comments and memory recall in both gardens
[40] White et al. (2018)	Pre-test post-test; RCT	1 year	Exposure to a nature garden	No control group	Mood	Datasheets	Logistic regression T1 > T0 (mean change score = 0.44, *p* < 0.001)
[42] Mitchell and Van Puymbroeck (2019)	Pre-test, post-test; single case	6 weeks	Therapeutic gardening and CBT 40–60 min, 3–4 times a week, over 6 weeks	No control group	Affect (anxiety)	BAI	Improvement 36%
					Depression	GDS-SF	Improvement 53%
					Falls	Number	Falls decreased from 7 to 0
[36] Pedrinolla et al. (2019)	Pre-test, post-test; RCT; single blind	6 months	Use of an indoor therapeutic garden (TG) 2 h per session, 5 times a week, 120 sessions N = 82	Spending time in a standard area (Control group, CG) N = 81	Behavior	NPI	Two-way repeated-measures ANOVA TG > CG in T1 (mean between groups difference of -31.8; 95% CI: -35.1 to -28.5; *F* = 279.2, *p* < 0.001)
					Cognition	MMSE	Two-way repeated-measures ANOVA TG > CG in T1 (mean difference between groups of 1.8; 95% CI: 1.4 to 2.2; *F* = 78.5, *p* < 0.001)
					Medications	Dosage of quetiapine	Two-way repeated-measures ANOVA TG > CG in T1 (−150 mg; 95% CI: −175 to −120: *F* = 87.3, *p* < 0.001
					Stress	Salivary cortisol	Two-way repeated-measures ANOVA TG > CG in T1 (*F* = 25.1, *p* < 0.001)
						Diastolic blood pressure	Two-way repeated-measures ANOVA (−2.6; mm Hg 95% CI: −3.5 to −1.7, *F* = 32.3, *p* < 0.001)
					Activity of the day	Barthel Index	Two-way repeated-measures ANOVA No difference between TG and CG in T1 (*F* = 2.1; ns)
[41] Collins et al. (2020)	Multiple treatment Single cases	12 weeks	Use of an indoor sensory garden and an outside sensory garden 30–45 min per session, 3 times a week	4 phases: baseline phase1, indoor sensory garden phase2, outside sensory garden phase3, return to baseline phase4	Agitation	CMAI, ABMI	phase2 and phase3 > phase1; phase3 > phase2
					Quality of life	DEMQOL, SIS	phase2 and phase3 > phase1; phase3 > phase2
[45] Gueib et al. (2020)	Pre-test, post-test	2 weeks	Use of a therapeutic garden (TG)12 h N = 16	No use of the therapeutic garden (Control group, CG)	Self-Consciousness	SCQ	TG > CG in T1 (TG: T1 SCQ = 10.41 [6.49–11.75] versus CG: T1 SCQ = 7.95 [6.00–9.16], *p* = 0.0079)

RCT: Randomized Controlled Trial; DCM: Dementia care mapping scale [48]; Short form CMAI: Short form Cohen Mansfield Agitation Inventory [51]; M-CMAI: Modified Cohen Mansfield Agitation Inventory [72]; HDS-R: Revised Hasegawa Dementia Scale [60]; AARS: Apparent affect Rating Scale [49]; MPES: Menorah Park Engagement Scale [47]; C-CMAI: Chinese version Cohen Mansfield Agitation Inventory; DEMQOL: Dementia Quality Of Life Instrument [54]; SCDD: Cornell Scale for Depression in Dementia [57]; MMSE: Mini Mental State Examination [59]; BAI: Beck Anxiety Inventory [50]; GDS-SF: Geriatric Depression Scale-Short Form [58]; NPI: Neuropsychiatric Inventory Scale [52]; ABMI: Agitated Behavior Mapping Instrument [53]; SIS: Six Item Screener [73]; SCQ: Self Consciousness Questionnaire [61,62,63]; WASO: wake up after sleep onset; NST: nocturnal sleep time; NSE: nocturnal sleep efficacy; TST: total sleep time; AE: active engagement; PE: passive engagement; SE: self-engagement; NE: non engagement; OE: other engagement; CBT: Cognitive Behavioral Therapy.
